# Iodine deficiency is associated with increased thyroid hormone sensitivity in individuals with elevated TSH

**DOI:** 10.1530/ETJ-21-0084

**Published:** 2022-03-23

**Authors:** Ying Sun, Di Teng, Lei Zhao, Xiaoguang Shi, Yongze Li, Zhongyan Shan, Weiping Teng

**Affiliations:** 1 Department of Endocrinology and Metabolism, Institute of Endocrinology, NHC Key Laboratory of Diagnosis and Treatment of Thyroid Diseases, The First Hospital of China Medical University, Shenyang, Liaoning, China; 2 Department of Endocrinology, Shengjing Hospital, China Medical University, Shenyang, Liaoning, China; 3 Department of Laboratory Medicine, The First Affiliated Hospital of China Medical University, Heping District, Shenyang, Liaoning, China

**Keywords:** iodine, thyroid hormone sensitivity, thyrotropin, thyroid feedback

## Abstract

**Objective:**

Central sensitivity of thyroid hormone refers to the sensitivity of hypothalamic–pituitary–thyroid (HPT) axis to the change in circulating free thyroxine (fT4). A complex relationship exists between thyroxine levels and iodine nutritional status. To explore the relationship between thyroid hormone sensitivity and iodine nutritional status in elevated thyrotropin (TSH), we used national data to assess the relationship between thyroid hormone sensitivity and iodine nutritional status with contrasting demographic characteristics in China.

**Methods:**

We enrolled 12,197 participants with TSH > 4.2 mIU/L from China. Serum and urine samples were collected, and we measured serum fT4, TSH, thyroid peroxidase antibody (TPOAb), and thyroglobulin antibody (TgAb) levels and urinary iodine concentration (UIC). The thyroid hormone sensitivity indices were calculated based on fT4 and TSH. The thyroid feedback quantile-based index (TFQI) is a new index to reflect thyroid hormone sensitivity. Higher TFQI quartiles indicated lower thyroid hormone sensitivity.

**Results:**

The odds ratios (ORs) for the fourth versus first TFQI quartile were 0.84 (95% CI 0.72–0.99) for iodine deficiency, 1.24 (95% CI 1.05–1.47) for TPOAb+, and 0.44 (95% CI 0.40–0.50) for females. The OR of the fourth and first TFQI quartiles for age <30 years and >60 years was 2.09 (95% CI 1.82–2.41) and 1.19 (95% CI 1.05–1.36), respectively (*P*  < 0.05). Other thyroid sensitivity indices also yielded similar results.

**Conclusion:**

Thyroid hormone sensitivity and age have a U-shaped association in individuals with elevated TSH. Increased thyroid hormone sensitivity is associated with iodine deficiency and the female gender. Decreased thyroid hormone sensitivity is associated with TPOAb+. These findings are interesting and potentially useful for understanding the interaction between iodine nutrition and the hypothalamic–pituitary–thyroid axis.

## Introduction

Recent evidence has suggested that impaired thyroid hormone (TH) sensitivity is associated with obesity, metabolic syndrome, diabetes, and diabetes-related mortality even in those with a normothyroid range (1). Aging modulates TH function, and elevated thyrotropin (TSH) in older adults is thought to help extend life (2). A recent study showed that levothyroxine (LT4) treatment for >1 year did not improve symptoms in patients aged >65 years with subclinical hypothyroidism (SCH) (3). Animal studies have shown that aging is associated with a low thyroid state and organ-specific sensitivity to thyroxine (4). Previous studies have suggested that age has an important influence on TH sensitivity (4). Iodine is the main component in TH synthesis, and it has additional thyroid biological functions. In iodine-deficient populations, increased iodine intake has been associated with a small increase in the prevalence of SCH and autoimmune thyroid diseases (AITDs) (5). Our previous epidemiological study showed that TSH levels in the population increased with the increase in iodine intake, and hypothyroidism and SCH incidence increased in iodine-deficient areas (6). The majority of patients with autoimmune thyroiditis have high concentrations of thyroid peroxidase antibodies (TPOAb) and anti-thyroglobulin antibodies (TgAb), which may have a specific impact on thyroid function (7). Generally, the prevalence of thyroid disease is higher in women than in men (8, 9).

The hypothalamic–pituitary–thyroid (HPT) axis is precisely regulated to maintain relatively constant TH levels during circulation. Peripheral tissues and the CNS control the availability of TH in cells, suggesting that the TH content in the circulation cannot fully reflect its role in the body. Some patients with hypothyroidism treated with LT4 have normal thyroid function but still show clinical symptoms, and animal studies pertaining to this have shown that the difference in deiodinase ubiquitination leads to differences in local TH sensitivity (10). The manifestations of TH sensitivity can be categorized as follows: (i) central sensitivity phenomena, which affect the feedback loop within the CNS and (ii) peripheral sensitivity phenomena, which decrease TH metabolic effects. Often, patients with central sensitivity also display TH sensitivity at the peripheral level. Generally, central resistance may be related to a general decrease in TH sensitivity, not only centrally but also in the periphery (1). In addition, patients with central sensitivity usually show peripheral TH insensitivity (11). However, the relationship between the thyroid function-related indicators and TH sensitivity remains unclear, and the current limited studies are based on patients with normal thyroid function. Therefore, abnormal TSH levels, especially regarding the relationship between elevated TSH status and TH sensitivity, are worth discussing. The thyroid feedback quantile-based index (TFQI) is a new index for assessing TH sensitivity and can quantify in a continuous manner the deviations from the median pituitary response (inhibition) to TH (1). Therefore, we analyzed a nationwide epidemiological survey of iodine nutrition status and thyroid disease to explore the characteristics of TH sensitivity and the related influencing factors in elevated TSH.

## Methods

### Study design, setting, and participants

**Figure 1:**
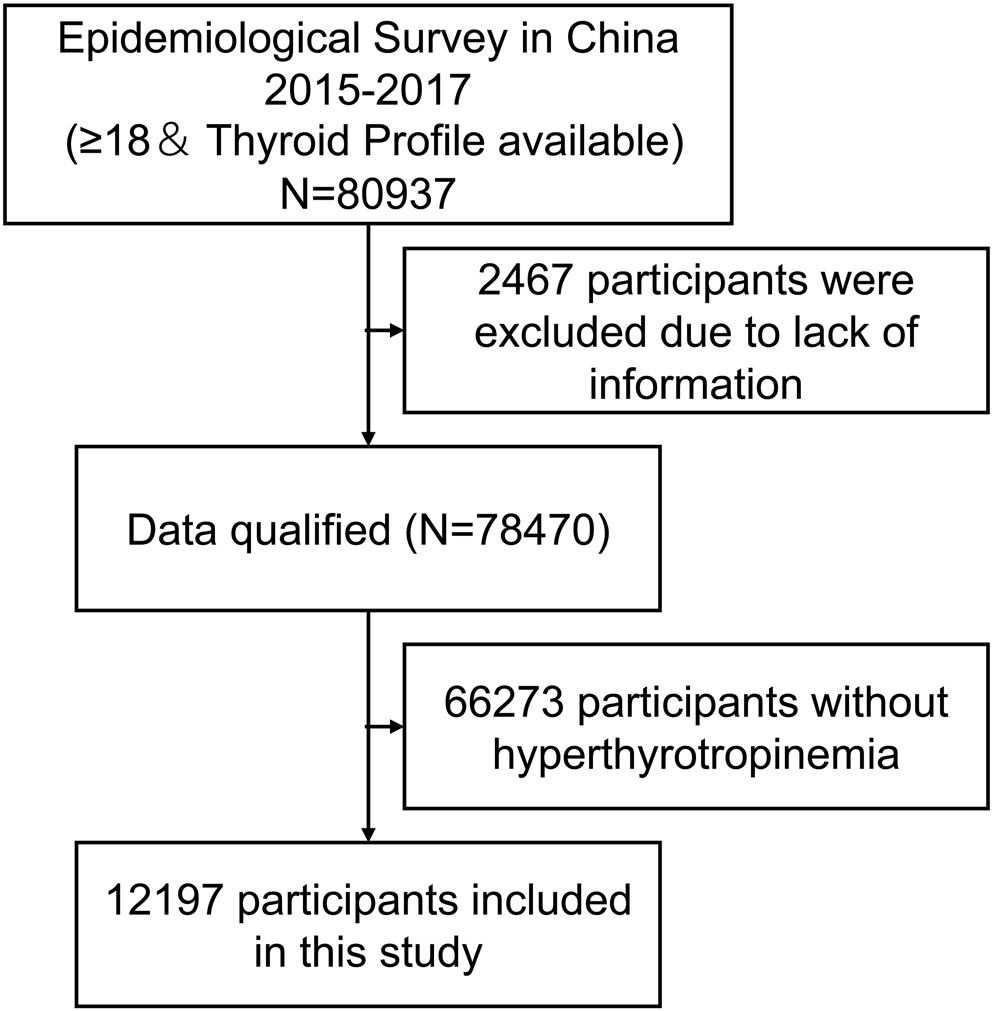
Study screening flowchart. The data for the present analysis were obtained from an epidemiological survey in China spanning 2015–2017. The aim of the nationwide survey was to evaluate the iodine nutrition status and thyroid disorders in those aged ≥18 years in China. We selected the elevated TSH population for the analysis.

The data used in the present study were extracted from thyroid disorders, iodine status, and the Diabetes Epidemiological Survey that had been conducted from June 2015 to June 2017 in China. We enrolled 31 provinces in mainland China using a whole-cluster, stratified random sampling design. Using the latest national census data, parallel random sampling was performed in rural locations based on the urban and rural sex–age ratios, with a total sample size of 80,937 people. The details on recruitment and data collection have been described previously (12, 13). After excluding 2467 people with missing crucial information and 66,273 people without elevated TSH, our analysis included 12,197 participants. The inclusion criteria were: (1) TSH ≥ 4.2 mIU/L; (2) not receiving thyroxine; (3) not pregnant; (4) age ≥ 18 years; (5) lived in the selected community for at least 5 years; (6) had not taken iodine-containing drugs or contrast agents in the past 3 months; (7) other known disease history had been excluded. The patient screening process is shown in Fig. 1.

### Data collection and laboratory measurements

The participants’ sociodemographic, regional location, lifestyle factor, and medical history data were collected with standardized questionnaires managed by professionally trained employees. The participants were asked to fast for 8 h overnight before their venous blood was collected. The blood and the urine samples were obtained from subjects at the same time. The blood samples were centrifuged, separated and then sent to the Shenyang Central Laboratory through the cold chain air transmission system for testing the thyroid parameters and urinary iodine concentration (UIC). The serum and urine samples were stored at −20°C. TSH, TPOAb, and TgAb levels were measured via electrochemiluminescence immunoassays with a Cobas 601 analyzer (Roche Diagnostic). Free thyroxine (fT4) and free triiodothyronine (fT3) were measured when the participant’s TSH levels were outside the reference limit (0.27–4.20 mIU/L). The normal reference ranges for thyroid function were, TSH, 0.27–4.2 mIU/L; fT4, 12.0–22.0 pmol/L; fT3, 3.1–6.8 pmol/L; TPOAb, <34.0 IU/mL; and TgAb, <115.0 IU/mL, as reported by the test kit manufacturers. The UIC was measured by inductively coupled plasma mass spectrometry (Agilent 7700x; Agilent Technologies). The results of UIC levels were shown as spot urine. Quality control was performed using the standard products GBW09108, GBW9109, and GBW9110 from the China Center for Disease Control and Prevention. The target values of the standards were TSH, 70.8 ± 9.0 μg/L; TPOAb, 143 ± 10 μg/L; and TgAb, 224 ± 14 μg/L. The intra-assay coefficients of variation (CVs) for TSH, TPOAb, and TgAb were 2.3%, 2.5%, and 2.4%, respectively; the intra-assay CVs were 2.7%, 1.4%, and 2.3%, respectively.

### Iodine group

Iodine status was based on the UIC and divided into four groups as established by guidelines from the World Health Organization: deficient iodine (DI UIC, <100 µg/L), adequate iodine (AI UIC, 100–199 µg/L), more than adequate iodine (MAI UIC, 200–299 µg/L), and excess iodine intake (EI UIC, ≥300 µg/L).

### TH sensitivity index

We used three indexes to evaluate the participants’ TH sensitivity. The TSH thyroxine sensitivity index (TT4RI) was calculated as: fT4 (pmol/L) **·** TSH (mIU/L) (11). The TSH index (TSHI) was calculated as: ln TSH (mIU/L) + 0.1345 **·** fT4 (pmol/L) (14). The TFQI is achieved by applying the population empirical cumulative distribution function (cdf) to hormone concentration: cdf fT4 − (1 − cdf TSH) (1).

The TFQI, TSHI, and TT4RI were regarded as the central indicators of TH sensitivity that reflected the sensitivity of the HPT axis to changes in circulating fT4. TFQI is a new index developed by researchers to quantify the deviation from median pituitary response to thyroid hormone in a continuous manner (1). The formula converts the grades of fT4 and TSH (in order from minimum to maximum) into quantiles between 0 and 1. This index was validated in a recent article on the relationship between TH sensitivity and diabetes mellitus (1). The TFQI based on the empirical joint distribution of fT4 and TSH has the advantage of not yielding extreme values in the case of thyroid gland dysfunction. These indices enabled us to study the pituitary feedback response with a continuous variable. All these TH sensitivity indices measure central sensitivity. The TFQI value is between −1 and 1. A positive value indicates that the HPT axis is less sensitive to changes in fT4. A value of 0 indicates that the HPT axis has normal sensitivity to changes in fT4. Negative values indicate that the HPT axis is more sensitive to changes in fT4. For the TSHI and TT4RI, a higher value indicates lower central sensitivity to TH.

### Statistical analysis

The mean number of thyroid parameters was calculated for sex, age, ethnicity, iodine status, and thyroid autoantibodies. Data were compared with independent *t* tests between two groups and with one-way ANOVA between all groups. The *P* values in Table 1 were calculated using linear regression analysis. Given their skewed distributions, the TSHI and TT4RI were processed in the logarithmic scale. Odds ratios (ORs) and 95% CIs were calculated by univariate and multivariate logistic regression to examine the association between the TH sensitivity index and iodine status. Model 1 was adjusted for sex, age, and ethnicity. Model 2 was adjusted for UIC. For all measures, a two-tailed *P*  < 0.05 was considered statistically significant, whereas an adjusted *P*  < 0.05 was applied for comparisons between different UICs. The above statistical analyses were performed using SPSS 22.0 (SPSS Inc., Chicago, IL, USA). Potential nonlinear associations between age and TH sensitivity were examined with restricted cubic splines, these analyses were performed with statistical computing software R.

**Table 1:** Thyroid parameters and population characteristics, sex, age, ethnicity, Iodine status, thyroid autoimmune antibodies, and smoking of Chinese elevated TSH population.

	%	FT4	*P*	TSH	*P*	TT4RI	*P*	TSHI	*p*	TFQI	*P*
All		15.53 (2.87)		7.67 (9.80)		96.77 (0.38)		3.90 (0.11)		0.00 (−0.23, 0.23)	
Sex											
Male	37.5	16.34	<0.05	7.32	<0.05	**99.13**	<0.05	**3.99**	<0.05	0.58 (−0.15, 0.31)	<0.05
Female	62.5	15.04		7.87		**95.38**		**3.87**		−0.02 (−0.26, 0.17)	
Age											
<30	20.9	16.70	<0.05	6.48	<0.05	**96.00**	<0.05	**3.98**	<0.05	0.06 (−0.16, 0.31)	<0.05
30–60	57.3	15.25		7.80		**95.67**		**3.87**		-0.01 (−0.25, 0.19)	
>60	21.8	15.12		8.48		**100.50**		**3.91**		0.01 (−0.21, 0.22)	
Ethinic											
Han	87.5	15.63	<0.05	7.60	<0.05	96.53	0.10	3.91	<0.05	0.01 (−0.22, 0.23)	<0.05
Uygurt	2.3	14.32		7.37		92.54		**3.78**		−0.07 (−0.28, 0.08)	
Hui	3.9	14.93		7.50		95.49		3.85		−0.01 (−0.21, 0.18)	
Zhuang	1.6	14.79		10.43		106.87		3.96		0.00 (−0.22, 0.18)	
Tibet	4.7	14.93		8.40		101.44		3.90		0.00 (−0.22, 0.22)	
Iodine											
DI	17.1	**15.13**	<0.05	8.09	0.13	96.59	0.43	**3.88**	<0.05	−0.04 (−0.28, 0.14)	<0.05
AI	37.4	15.55		7.59		96.60		3.90		−0.01 (−0.25, 0.19)	
MAI	21.8	15.70		7.60		97.24		3.92		0.00 (−0.22, 0.24)	
EI	23.2	15.66		7.49		96.67		3.91		0.01 (−0.22,0.24)	
Antibody											
TPOAb+	22.2	14.28	<0.05	11.48	<0.05	**110.90**	<0.05	**3.96**	<0.05	0.01 (−0.24, 0.24)	0.93
TPOAb−	77.8	15.89		6.58		**93.08**		**3.89**		0.00 (−0.18, 0.18)	
TgAb+	20.6	14.31	<0.05	10.92	<0.05	**108.14**	<0.05	**3.94**	<0.05	0.01 (−0.23, 0.24)	0.13
TgAb−	79.4	15.85		6.83		**94.02**		**3.90**		0.00 (−0.20, 0.18)	
Smocking											
Yes	17.6	16.17	<0.05	7.76	0.64	**99.97**	<0.05	**3.99**	<0.05	0.04 (−0.24, 0.22)	<0.05
No	82.2	15.40		7.65		**96.13**		**3.89**		0.00 (−0.15, 0.30)	

Data are percentages or means (s.d.) unless TFQI. The data of TFQI are median plus interquartile range. *P* values are for differences between groups and were calculated from linear regression model.

Bold indicates statistical significance.

## Results

**Figure 2:**
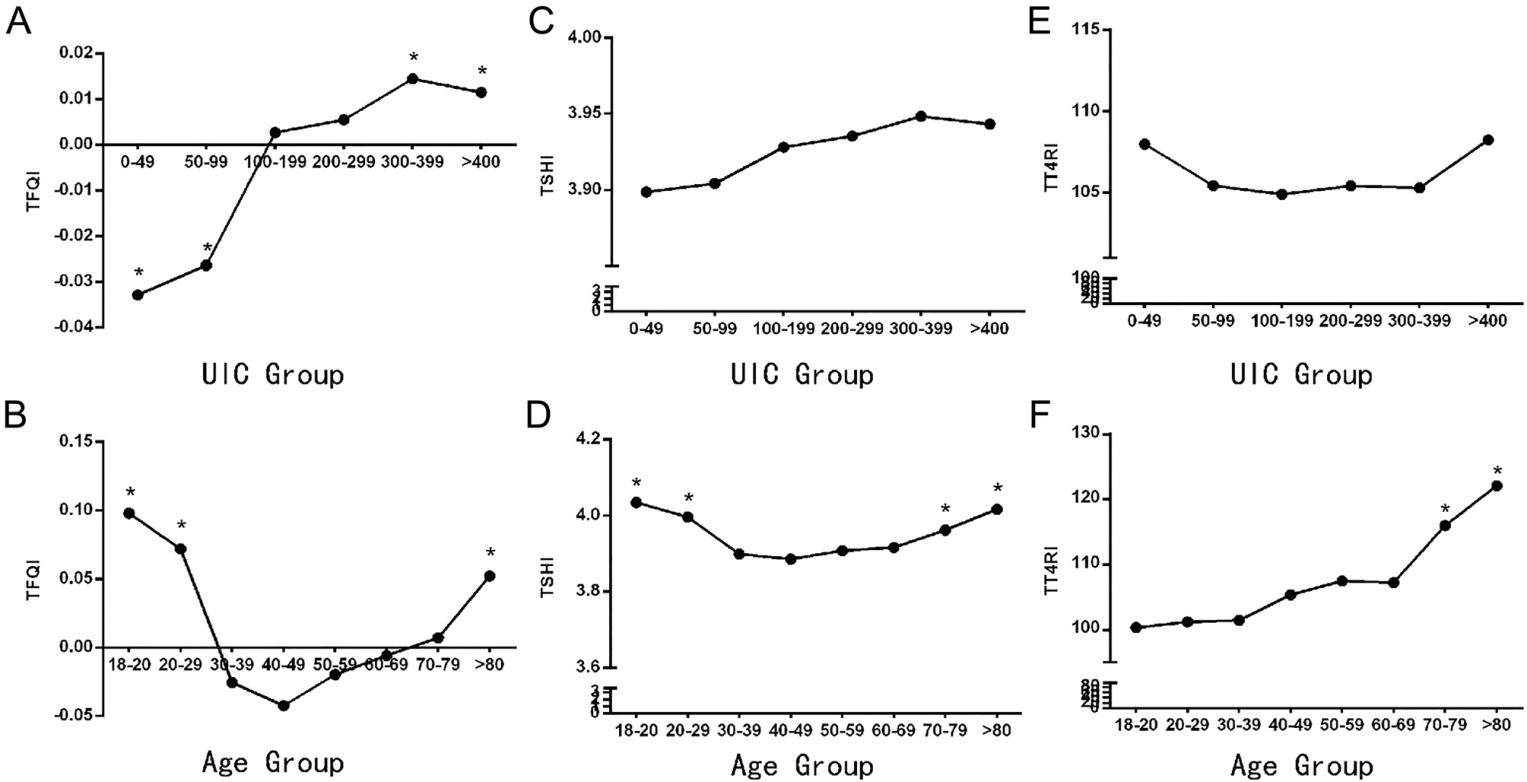
Association of the TFQI, TSHI, and TT4RI with different UIC and age groups. (A and B) The overall TFQI stratified by UIC and age. *P  < 0.05. (C and D) The overall TSHI stratified by UIC and age. *P  < 0.05. (E and F) The overall TT4RI stratified by UIC and age. *P  < 0.05.

The 12,197 participants in the present study represented the Chinese population aged ≥18 years, with TSH levels greater than the upper limit of the reference value (4.2 mIU/L) (Fig. 1). The general characteristics and TH sensitivity indices of the study population are presented in Table 1. TH sensitivity was higher among women and the Uygur ethnic group and was lower among those with positive thyroid autoantibodies and smoking habits (Table 1). There is a nonlinear relationship between age and TH sensitivity, and iodine deficiency (<100 μg/L) was associated with increased TH sensitivity (Fig. 2 and Table 1).

We calculated the ORs for iodine, thyroid autoantibodies, and each of the criteria across the TFQI quartiles using logistic models. Model 1 was adjusted for sex, age, and ethnics, and the OR of the fourth vs first TFQI quartile for the UIC (<100 μg/L) was 0.84 (95% CI 0.72–0.99) (*P*  < 0.05). Model 2 was further adjusted for UIC, and the OR of the fourth, third, and second TFQI quartiles vs the first TFQI quartile for TPOAb was 1.48 (95% CI 1.27–1.73), 1.55 (95% CI 1.33–1.81), and 1.24 (95% CI 1.05–1.47), respectively (*P*  < 0.05) (Table 2).

**Table 2:** Association of TFQI with sex, age, iodine, thyroid antibody in the sample.

	TFQI			
	Quartile 1 (≥−1, and <−0.25)	Quartile 2 (≥−0.25, and<0)	Quartile 3 (≥0, and<0.25)	Quartile 4 (≥−0.25, and≤0)
*N*	2796	3206	3352	2842
Model 1, difference (95% CI)				
UIC (µg/L)				
<100	1.00 (reference)	0.92 (0.79–1.06)	0.98 (0.85–1.14)	**0.84 (0.72–0.99)**
100–199	-	-	-	-
200–299	1.00 (reference)	0.96 (0.84–1.10)	1.05 (0.92–1.20)	0.91 (0.79–1.05)
≥300	1.00 (reference)	0.91 (0.79–1.05)	1.06 (0.92–1.21)	0.92 (0.80–1.07)
Model 2, difference (95% CI)				
Antibody				
TPOAb−	-	-	-	-
TPOAb+	1.00 (reference)	**1.48 (1.27–1.73)**	**1.55 (1.33–1.81)**	**1.24 (1.05–1.47)**
TgAb−	-	-	-	-
TgAb+	1.00 (reference)	1.11 (0.94–1.29)	0.99 (0.85–1.56)	0.98 (0.82-1.56)

Differences in ORs are estimated with logistic regression model. Model 1 was adjusted for sex, age, and ethnics. Model 2 was further adjusted for UIC.

Bold indicates statistical significance.

We also calculated the OR using the TSHI. The association was independent of autoantibodies after adjustment for sex, age, UIC, and ethnicity. The OR of the fourth vs first TSHI quartile for TPOAb and TgAb was 1.67 (95% CI 1.48–1.88) and 1.46 (95% CI 1.29–1.65), respectively (*P*  < 0.05) (Table 3).

**Table 3:** Association of TSHI with sex, age, iodine, thyroid antibody in the sample.

	TSHI			
	Quartile 1 (lo.,3.63)	Quartile 2 (3.63,3.88)	Quartile 3 (3.88,4.17)	Quartile 4 (4.17,hi.)
N	3042	3027	3031	3030
Model 1, difference (95% CI)				
UIC (µg/L)				
<100	1.00 (reference)	0.90 (0.78–1.05)	1.00 (0.86–1.62)	0.98 (0.85–1.41)
100–199	-	-	-	-
200–299	1.00 (reference)	0.96 (0.84–1.10)	0.97 (0.85–1.11)	0.96 (0.86–1.13)
≥300	1.00 (reference)	0.97 (0.84–1.13)	0.93 (0.81–1.07)	1.00 (0.87–1.15)
Model 2, difference (95% CI)				
Antibody				
TPOAb−	-	-	-	-
TPOAb+	1.00 (reference)	0.97 (0.85–1.10)	1.09 (0.96–1.23)	**1.67 (1.48–1.88)**
TgAb−	-	-	-	-
TgAb+	1.00 (reference)	0.93 (0.82–1.06)	0.99 (0.87–1.22)	**1.46 (1.29–1.65)**

Differences ORs are estimated with logistic regression model. Model 1 was adjusted for sex, age, and ethnics. Model 2 was further adjusted for UIC.

Bold indicates statistical significance.

Further adjustment for sex, age, UIC, and ethnicity calculated using the TT4RI, the OR of the fourth vs first TT4RI quartile for TPOAb and TgAb was 2.16 (95% CI 1.86–2.51) and 1.36 (95% CI 1.17–1.59), respectively (*P*  < 0.05). The OR of the third vs first TT4RI quartile for TPOAb was 1.19 (95% CI 1.02–1.40) (Table 4).

**Table 4:** Association of TT4RI with sex, age, iodine, thyroid antibody in the sample.

	TT4RI			
	Quartile 1 (lo.,74.80)	Quartile 2 (74.80,88.31)	Quartile 3 (88.31,111.53)	Quartile 4 (111.53,hi.)
N	3042	3027	3031	3030
Model 1, difference (95% CI)				
UIC (µg/L)				
<100	1.00 (reference)	0.84 (0.72–0.97)	0.88 (0.76–1.02)	0.94 (0.81–1.09)
100–199	-	-	-	-
200–299	1.00 (reference)	0.92 (0.80–1.05)	0.96 (0.84–1.10)	0.97 (0.85–1.11)
≥300	1.00 (reference)	0.88 (0.77–1.01)	0.91 (0.79–1.04)	0.95 (0.83–1.09)
Model 2, difference (95% CI)				
Antibody				
TPOAb−	-	-	-	-
TPOAb+	1.00 (reference)	1.15 (0.98–1.35)	**1.19 (1.02–1.40)**	**2.16 (1.86–2.51)**
TgAb−	-	-	-	-
TgAb+	1.00 (reference)	0.97 (0.82–1.14)	1.02 (0.87–1.20)	**1.36 (1.17–1.59)**

Differences in ORs are estimated with logistic regression model. Model 1 was adjusted for sex, age, and ethnics. Model 2 was further adjusted for UIC.

Bold indicates statistical significance.

**Figure 3:**
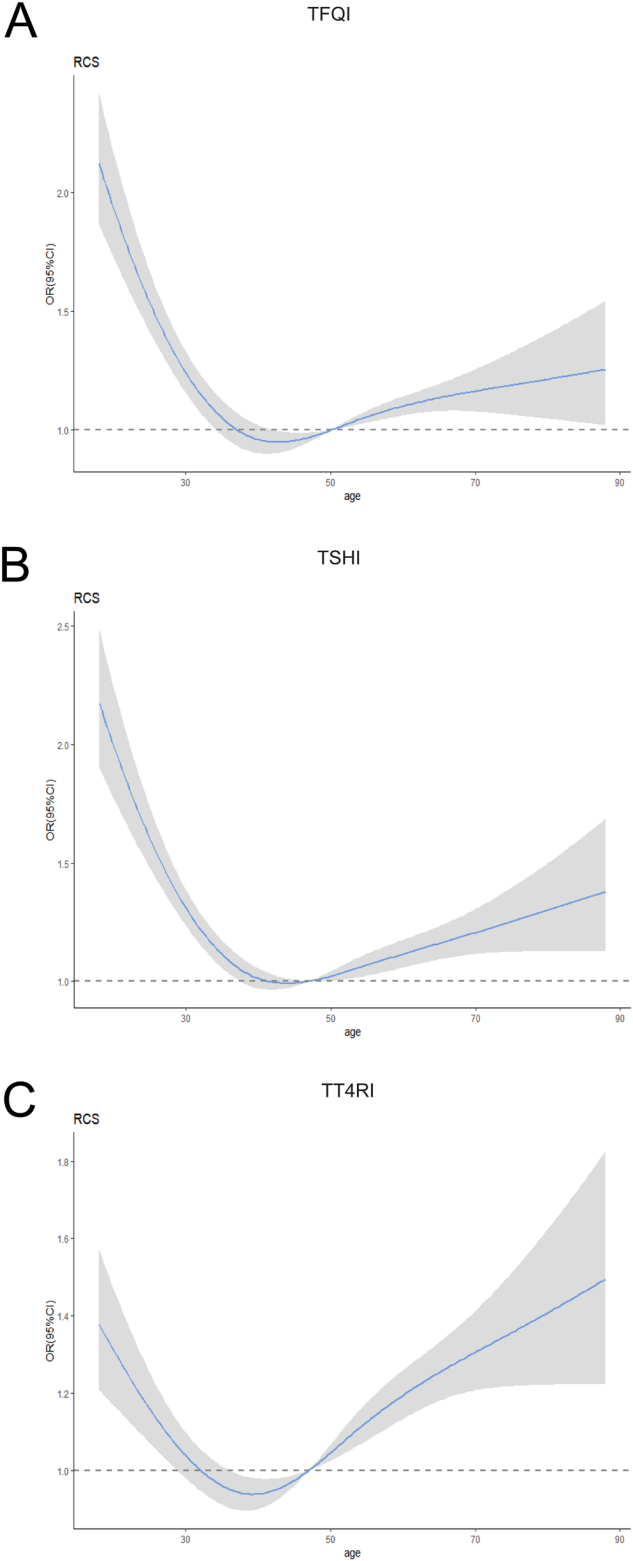
Correlation coefficient and 95%CI for age and thyroid hormone sensitivity. The solid line is the correlation coefficient adjusted for sex, and the shaded area indicates that 95% CI is derived from restricted cubic spline regression.

Restricted cubic spline analyses suggested an association between age and TH sensitivity that younger and older age were associated with decreased TH sensitivity (Fig. 3).

## Discussion

The relationship between TSH levels and iodine continues to be a concern. In addition, the establishment of a reference range and treatment of SCH and hypothyroidism have been controversial (7, 15). However, the diagnosis of subclinical thyroid diseases depends on the sensitivity of the HPT axis (15). The present study was based on TIDE (Thyroid disease, Iodine nutrition, and Diabetes Epidemiology) study data, which are representative of the Chinese elevated TSH population. We provide evidence for the association between the TH sensitivity indices and sex, age, iodine nutritional status, TPOAb, and TgAb. These associations represent the characteristics of patients with elevated TSH and have shown importance in hypothyroidism and SCH treatment strategies.

We have included the TH sensitivity indicators proposed by Laclaustra *et al.* (1), and the present study is the first to use elevated TSH population to evaluate TH sensitivity and iodine nutritional status. We show that in a elevated TSH population, women have higher TH sensitivity than men. Both younger (<30 years) and older age (>60 years) were associated with decreased TH sensitivity. Iodine deficiency (UIC < 99 μg/L) was associated with increased TH sensitivity. Patients with positive antibodies within the elevated TSH population had decreased TH sensitivity.

Hypothyroidism is common worldwide. Previous studies have generally believed that the female gender is a risk factor for thyroid disease. The incidence of hypothyroidism is approximately 10 times higher in women than in men (9). Some studies have suggested that the sex hormones and inactivation of the X chromosome may be the cause of hypothyroidism and hyperthyroidism (16). In the present study, we showed that TH sensitivity is higher in the female elevated TSH population, suggesting that women may be more likely to benefit from hypothyroidism treatment, but this is only theoretical. This relationship requires verification by further studies.

Previous studies have found that aging is related to elevated serum TSH levels (17, 18, 19). Treatment for SCH in older patients remains controversial. A previous 4-year follow-up study suggested that 85–89-year-old patients with hypothyroidism and SCH have a longer life span and lower cardiovascular mortality rate (20). Bekkering *et al.* indicated that there is almost no difference in general quality of life (QoL), thyroid-related symptoms, depressive symptoms, fatigue, cognitive function, muscle strength, and BMI in treated vs untreated older people with SCH (21). Another study found that LT4 had no obvious benefit to elderly patients with SCH (22, 23). In the present study, we found that older age (>60 years) was associated with decreased TH sensitivity. This result may provide evidence for poor treatment in elderly patients with SCH. There are few randomized controlled trials with LT4 replacement therapy for SCH in young patients (24, 25). Furthermore, there is limited evidence for the possible benefits and risks of treatment, and previous studies in the younger patient age group have been insufficiently detailed (26). The present study shows that younger patients (age < 30 years) within the elevated TSH population had decreased TH sensitivity. Our results show that there is an urgent need for careful testing of SCH and that clinical trials should be larger than previous studies, focusing on young patients and controlling for other factors.

Iodine is an important trace element for TH synthesis, and a complicated relationship exists between iodine intake and thyroid diseases. The relationship between iodine nutritional status and thyroid diseases of the population is U-shaped because insufficient and excessive iodine intake damages thyroid function (5). In patients with previous iodine deficiency, a small increase in iodine intake changed the pattern of thyroid disease (27). Severe iodine deficiency can cause goiter and hypothyroidism. In mild to moderate iodine deficiency, increased thyroid activity can compensate for iodine deficiency in most patients and maintain normal thyroid function. However, chronic thyroid stimulation can lead to increased prevalence of nodular goiter and hyperthyroidism in the population. Therefore, increasing the iodine intake would usually lead to a further increase in the prevalence of hyperthyroidism (28, 29). In a previous 5-year prospective follow-up study, we found that there was no significant difference in hypothyroidism incidence between areas with moderate iodine deficiency and iodine excess, but SCH incidence was increased in areas with iodine excess, and the incidence was 0.2 and 2.9%, respectively (6). Whether the effects of iodine in thyroid diseases are transient or lasting, and the mechanism of action, remains unclear. Studies on the relationship between TH sensitivity and iodine intake have shown that iodine deficiency is related to increased TH sensitivity. This result may explain the increased hyperthyroidism incidence caused by iodine supplementation after iodine deficiency, and the increased prevalence of SCH due to iodine supplementation. The TFQI is based on the empirical joint distribution of fT4 and TSH and has the advantage of not yielding extreme values in cases of thyroid gland dysfunction (1). Although this association was strong enough to yield statistically significant results in the TFQI, we found no statistical difference between the other two indexes. Therefore, more confirmatory research is needed.

Thyroid autoantibodies, that is, TPOAb and TgAb, are commonly found in the serum of patients with AITD and have important effects on thyroid function. In 1999 and 2011, we showed that the positive rate of TPOAb in the general Chinese population was 9.81 and 11.5%, respectively, and that for TgAb was 9.09 and 12.6%, respectively (6, 30). In the present study, we found that thyroid autoantibodies, especially TPOAb, were related to reduced TH sensitivity.

In addition, we showed that the Uyghur ethnic group is more sensitive to TH and that people who smoke are less sensitive to TH. Previous studies have shown that Blacks are more sensitive to TH (1). Additionally, there is some evidence that ethnicity has an effect on thyroid function (31), such as the prevalence of hypothyroidism being lower in Blacks (32). Furthermore, smoking has a specific impact on thyroid function (33).

The HPT axis accurately regulates TH synthesis and secretion. Existing research shows that TH transporters, deiodinases, and TH receptor coregulators can regulate TH tissue sensitivity (34). In hypothyroidism, the coordinated expression and activity of deiodinase regulates TH levels. Patients with central sensitivity usually show TH insensitivity at the peripheral level (11). Despite proper treatment, clinical symptoms persist in approximately 15% of hypothyroidism patients treated with LT4 (35). Whether this phenomenon is related to decreased TH sensitivity warrants further study. Some studies have shown that not all patients may find LT4 therapy sufficient. Therefore, symptoms may be caused by residual hypothyroidism. In addition, the difference in the ubiquitination of hypothalamic type 2 deiodinase leads to the change in the local sensitivity of thyroxine (10, 36). Triiodothyronine is based on TH biological activity, and the decrease in TH sensitivity is mainly reflected in the sensitivity to thyroxine. Although randomized controlled clinical trials have compared the efficacy of LT4 monotherapy and LT4 plus triiodothyronine combination therapy for hypothyroidism, most of these trials found that despite the elevated serum triiodothyronine in combination-treated patients, both treatments had similar efficacy (37). However, further study is needed to show that combination therapy can benefit patients with poor TH sensitivity.

Our study has several limitations and constraints. First, we conducted large-scale nationwide epidemiological survey; the analysis was based on a single blood test, which is a common limitation in population-based studies. Although the questionnaire excluded the history of known non-thyroid diseases, there is still no guarantee that there were undetected non-thyroid diseases. As far as we are aware, this is the first time the indices of TH sensitivity have been evaluated in elevated TSH. This result is interesting but is nevertheless preliminary and should be confirmed with more research.

This study shows for the first time a cross-sectional association between TH sensitivity and iodine nutritional status in elevated TSH. One highlight of this study is the application of a new TH sensitivity index, that is, the TFQI. This index may be helpful for evaluating HPT axis regulation under physiologic or pathologic conditions. In the present study, sex, age, ethnicity, smoking, iodine deficiency, and thyroid autoantibodies were associated with TH sensitivity in patients with elevated TSH. These findings are interesting and potentially useful for understanding the interaction between iodine nutrition and the HPT axis.

## Declaration of interest

The authors declare that there is no conflict of interest that could be perceived as prejudicing the impartiality of the research reported.

## Funding

The study was funded by the Research Fund for Public Welfare, National Health and Family Planning Commission of China (Grant No. 201402005) and the National Natural Science Foundation of China (No. 81970682, 81800700), Liaoning Education Department project (JCZR2020005).

## Statement of ethics

Our research protocol was approved by the Medical Ethics Committee of the China Medical University. All participants provided written informed consent after receiving a detailed explanation of the research procedures.

## Data availability statement

The data that support the findings of this study are not publicly available due to we promised that the data will not be provided to the third parties when reviewed by the ethics committee. But if there are reasonable request, please ask the corresponding author.

## Author contribution statement

W P T and Z Y S conceived of and designed the study; Y Z L and D T organized and supervised the study; Y S and L Z conducted the statistical analysis. All authors contributed to the acquisition, analysis, or interpretation of data. W P T and Y S drafted the manuscript. All authors revised the report and approved of the final version before submission.

## References

[1] Laclaustra M Moreno-Franco B Lou-Bonafonte JM Mateo-Gallego R Casasnovas JA Guallar-Castillon P Cenarro A Civeira F. Impaired sensitivity to thyroid hormones is associated with diabetes and metabolic syndrome. Diabetes Care 2019 42 303–310. (10.2337/dc18-1410) 30552134

[2] Bowers J Terrien J Clerget-Froidevaux MS Gothié JD Rozing MP Westendorp RG van Heemst D Demeneix BA. Thyroid hormone signaling and homeostasis during aging. Endocrine Reviews 2013 34 556–589. (10.1210/er.2012-1056) 23696256

[3] Stott DJ Rodondi N Kearney PM Ford I Westendorp RGJ Mooijaart SP Sattar N Aubert CE Aujesky D Bauer DC Thyroid hormone therapy for older adults with subclinical hypothyroidism. New England Journal of Medicine 2017 376 2534–2544. (10.1056/NEJMoa1603825) 28402245

[4] Rakov H De Angelis M Renko K Hönes GS Zwanziger D Moeller LC Schramm KW Führer D. Aging is associated with low thyroid state and organ-specific sensitivity to thyroxine. Thyroid 2019 29 1723–1733. (10.1089/thy.2018.0377) 31441387

[5] Zimmermann MB Boelaert K. Iodine deficiency and thyroid disorders. Lancet: Diabetes and Endocrinology 2015 3 286–295. (10.1016/S2213-8587(1470225-6) 25591468

[6] Teng W Shan Z Teng X Guan H Li Y Teng D Jin Y Yu X Fan C Chong W Effect of iodine intake on thyroid diseases in China. New England Journal of Medicine 2006 354 2783–2793. (10.1056/NEJMoa054022) 16807415

[7] Chaker L Bianco AC Jonklaas J Peeters RP. Hypothyroidism. Lancet 2017 390 1550–1562. (10.1016/S0140-6736(1730703-1) 28336049 PMC6619426

[8] Aoki Y Belin RM Clickner R Jeffries R Phillips L Mahaffey KR. Serum TSH and total T4 in the United States population and their association with participant characteristics: National Health and Nutrition Examination Survey (NHANES 1999–2002). Thyroid 2007 17 1211–1223. (10.1089/thy.2006.0235) 18177256

[9] Taylor PN Albrecht D Scholz A Gutierrez-Buey G Lazarus JH Dayan CM Okosieme OE. Global epidemiology of hyperthyroidism and hypothyroidism. Nature Reviews: Endocrinology 2018 14 301–316. (10.1038/nrendo.2018.18) 29569622

[10] Werneck de Castro JP Fonseca TL Ueta CB McAninch EA Abdalla S Wittmann G Lechan RM Gereben B Bianco AC. Differences in hypothalamic type 2 deiodinase ubiquitination explain localized sensitivity to thyroxine. Journal of Clinical Investigation 2015 125 769–781. (10.1172/JCI77588) 25555216 PMC4319436

[11] Ercan-Fang S Schwartz HL Mariash CN Oppenheimer JH. Quantitative assessment of pituitary resistance to thyroid hormone from plots of the logarithm of thyrotropin versus serum free thyroxine index. Journal of Clinical Endocrinology and Metabolism 2000 85 2299–2303. (10.1210/jcem.85.6.6625) 10852467

[12] Li Y Teng D Ba J Chen B Du J He L Lai X Teng X Shi X Li Y Efficacy and safety of long-term universal salt iodization on thyroid disorders: epidemiological evidence from 31 provinces of Mainland China. Thyroid 2020 30 568–579. (10.1089/thy.2019.0067) 32075540

[13] Li Y Teng D Shi X Qin G Qin Y Quan H Shi B Sun H Ba J Chen B Prevalence of diabetes recorded in mainland China using 2018 diagnostic criteria from the American Diabetes Association: National Cross Sectional Study. BMJ 2020 369 m997. (10.1136/bmj.m997) PMC7186854 32345662

[14] Zipf G Chiappa M Porter KS Ostchega Y Lewis BG Dostal J. National health and nutrition examination survey: plan and operations, 1999–2010. Vital and Health Statistics, Series 1: Programs and Collection Procedures 2013 56 1–37. 25078429

[15] Cooper DS Biondi B. Subclinical thyroid disease. Lancet 2012 379 1142–1154. (10.1016/S0140-6736(1160276-6) 22273398

[16] Garmendia Madariaga A Santos Palacios S Guillén-Grima F Galofré JC. The incidence and prevalence of thyroid dysfunction in Europe: a meta-analysis. Journal of Clinical Endocrinology and Metabolism 2014 99 923–931. (10.1210/jc.2013-2409) 24423323

[17] Bremner AP Feddema P Leedman PJ Brown SJ Beilby JP Lim EM Wilson SG O’Leary PC Walsh JP. Age-related changes in thyroid function: a longitudinal study of a community-based cohort. Journal of Clinical Endocrinology and Metabolism 2012 97 1554–1562. (10.1210/jc.2011-3020) 22344200

[18] Waring AC Arnold AM Newman AB Bùzková P Hirsch C Cappola AR. Longitudinal changes in thyroid function in the oldest old and survival: the cardiovascular health study all-stars study. Journal of Clinical Endocrinology and Metabolism 2012 97 3944–3950. (10.1210/jc.2012-2481) 22879629 PMC3485600

[19] Surks MI Hollowell JG. Age-specific distribution of serum thyrotropin and antithyroid antibodies in the US population: implications for the prevalence of subclinical hypothyroidism. Journal of Clinical Endocrinology and Metabolism 2007 92 4575–4582. (10.1210/jc.2007-1499) 17911171

[20] Gussekloo J van Exel E de Craen AJ Meinders AE Frölich M Westendorp RG. Thyroid status, disability and cognitive function, and survival in old age. JAMA 2004 292 2591–2599. (10.1001/jama.292.21.2591) 15572717

[21] Bekkering GE Agoritsas T Lytvyn L Heen AF Feller M Moutzouri E Abdulazeem H Aertgeerts B Beecher D Brito JP Thyroid hormones treatment for subclinical hypothyroidism: a clinical practice guideline. BMJ 2019 365 l2006. (10.1136/bmj.l2006) 31088853

[22] Weickert MO Kyrou I. Thyroid hormone therapy for older adults with subclinical hypothyroidism. New England Journal of Medicine 2017 377 e20. (10.1056/NEJMc1709989) 29019378

[23] Mooijaart SP Du Puy RS Stott DJ Kearney PM Rodondi N Westendorp RGJ den Elzen WPJ Postmus I Poortvliet RKE van Heemst D Association between levothyroxine treatment and thyroid-related symptoms among adults aged 80 years and older with subclinical hypothyroidism. JAMA 2019 322 1977–1986. (10.1001/jama.2019.17274) 31664429 PMC6822162

[24] Akhtar MA Agrawal R Brown J Sajjad Y Craciunas L. Thyroxine replacement for subfertile women with euthyroid autoimmune thyroid disease or subclinical hypothyroidism. Cochrane Database of Systematic Reviews 2019 6 CD011009. (10.1002/14651858.CD011009.pub2) 31236916 PMC6591496

[25] Mikhail GS Alshammari SM Alenezi MY Mansour M Khalil NA. Increased atherogenic low-density lipoprotein cholesterol in untreated subclinical hypothyroidism. Endocrine Practice 2008 14 570–575. (10.4158/EP.14.5.570) 18753099

[26] Rugge JB Bougatsos C Chou R. Screening and treatment of thyroid dysfunction: an evidence review for the U.S. Preventive Services Task Force. Annals of Internal Medicine 2015 162 35–45. (10.7326/M14-1456) 25347444

[27] Laurberg P Jørgensen T Perrild H Ovesen L Knudsen N Pedersen IB Rasmussen LB Carlé A Vejbjerg P. The Danish investigation on iodine intake and thyroid disease, DanThyr: status and perspectives. European Journal of Endocrinology 2006 155 219–228. (10.1530/eje.1.02210) 16868134

[28] Laurberg P Pedersen KM Vestergaard H Sigurdsson G. High incidence of multinodular toxic goitre in the elderly population in a low iodine intake area vs. high incidence of Graves’ disease in the young in a high iodine intake area: comparative surveys of thyrotoxicosis epidemiology in East-Jutland Denmark and Iceland. Journal of Internal Medicine 1991 229 415–420. (10.1111/j.1365-2796.1991.tb00368.x) 2040867

[29] Krohn K Führer D Bayer Y Eszlinger M Brauer V Neumann S Paschke R. Molecular pathogenesis of euthyroid and toxic multinodular goiter. Endocrine Reviews 2005 26 504–524. (10.1210/er.2004-0005) 15615818

[30] Shan Z Chen L Lian X Liu C Shi B Shi L Tong N Wang S Weng J Zhao J Iodine status and prevalence of thyroid disorders after introduction of mandatory universal salt iodization for 16 years in China: a cross-sectional study in 10 cities. Thyroid 2016 26 1125–1130. (10.1089/thy.2015.0613) 27370068

[31] McLeod DS Caturegli P Cooper DS Matos PG Hutfless S. Variation in rates of autoimmune thyroid disease by race/ethnicity in US military personnel. JAMA 2014 311 1563–1565. (10.1001/jama.2013.285606) 24737370

[32] Sichieri R Baima J Marante T de Vasconcellos MT Moura AS Vaisman M. Low prevalence of hypothyroidism among black and Mulatto people in a population-based study of Brazilian women. Clinical Endocrinology 2007 66 803–807. (10.1111/j.1365-2265.2007.02816.x) 17381480

[33] Asvold BO Bjøro T Nilsen TI Vatten LJ. Tobacco smoking and thyroid function: a population-based study. Archives of Internal Medicine 2007 167 1428–1432. (10.1001/archinte.167.13.1428) 17620538

[34] Mendoza A Hollenberg AN. New insights into thyroid hormone action. Pharmacology and Therapeutics 2017 173 135–145. (10.1016/j.pharmthera.2017.02.012) 28174093 PMC5407910

[35] Saravanan P Visser TJ Dayan CM. Psychological well-being correlates with free thyroxine but not free 3,5,3′-triiodothyronine levels in patients on thyroid hormone replacement. Journal of Clinical Endocrinology and Metabolism 2006 91 3389–3393. (10.1210/jc.2006-0414) 16804044

[36] Jo S Fonseca TL Bocco BMLC Fernandes GW McAninch EA Bolin AP Da Conceição RR Werneck-de-Castro JP Ignacio DL Egri P Type 2 deiodinase polymorphism causes ER stress and hypothyroidism in the brain. Journal of Clinical Investigation 2019 129 230–245. (10.1172/JCI123176) 30352046 PMC6307951

[37] Grozinsky-Glasberg S Fraser A Nahshoni E Weizman A Leibovici L. Thyroxine-triiodothyronine combination therapy versus thyroxine monotherapy for clinical hypothyroidism: meta-analysis of randomized controlled trials. Journal of Clinical Endocrinology and Metabolism 2006 91 2592–2599. (10.1210/jc.2006-0448) 16670166

